# Editorial: African swine fever virus (ASFV) in the one health approach

**DOI:** 10.3389/fvets.2025.1675472

**Published:** 2025-08-29

**Authors:** Francesca De Falco

**Affiliations:** Area Science Park, Trieste, Italy

**Keywords:** African swine fever, epidemiology, virus, economic impact, genetic complexity

African Swine Fever (ASF) remains a critical threat to pig populations globally. The disease, caused by the African Swine Fever Virus (ASFV), has devastating effects on animal health, the swine industry, and the livelihoods of millions who rely on pig farming. With no commercially available vaccine and a complex epidemiology involving both domestic pigs and wild suids, ASF continues to be a top priority for researchers, veterinarians, and animal health authorities worldwide.

This Research Topic compiles nine recent studies that deepen our understanding of ASFV, enhance diagnostics and control, and explore the broader implications of outbreaks across different sectors.

An often-overlooked aspect of animal disease outbreaks is their impact on the mental health of responders. In this context, Bakke et al. examine the psychological toll on veterinarians involved in ASF control in the Philippines. The authors highlight the urgent need for psychological support, including access to counseling and training in emotional resilience, within outbreak response frameworks. Their findings highlight the emotional strain associated with disease management and call for the inclusion of mental health support in veterinary response planning.

Several studies focus on improving ASFV diagnostics. Zhang et al. describe a novel triple protein-based ELISA that improves sensitivity in antibody detection. Hu et al. introduce a duplex fluorescent qPCR assay capable of distinguishing genotype I, II, and recombinant strains currently circulating in China. Complementing these laboratory advances, Li et al. conduct a retrospective field study evaluating optimal sampling strategies for ASF surveillance.

On the biosecurity front, Hemmink et al. explore environmental mitigation tools, reporting virus-inactivating properties of rosin-functionalized plastic surfaces. This finding opens the door to novel materials that may enhance on-farm biosecurity practices (Hemmink et al.).

Feed safety is another area of interest, particularly due to concerns about indirect virus transmission. Shurson et al. assess the persistence of an ASF-like surrogate algal virus in feed ingredients under storage and digestion conditions, offering insights into feed-related risks and mitigation strategies.

Surveillance systems also benefit from novel data sources. Hsu et al. demonstrate that Google Trends can act as an early warning tool for ASF outbreaks in Southeast Asia, suggesting a role for digital surveillance to complement conventional approaches.

Spatial analysis supports targeted control strategies. Ko et al. apply a statistical model to identify ASF risk clusters in Korea, providing valuable guidance for regional planning and response.

Finally, Alotaibi et al. offer a broad review of ASFV reservoirs, transmission pathways, and genomic characteristics. Their work highlights gaps in wildlife surveillance and emphasizes the need for international cooperation in monitoring and control (Alotaibi et al.).

## Collective insights and the path forward

Together, the studies presented in this Research Topic underscore the complexity of ASF and the need for interdisciplinary approaches. Key themes that emerge include:

- Enhanced diagnostics: new tools such as improved ELISAs and genotype-specific qPCRs are helping detect infections more accurately and quickly, supporting early intervention.
- Field sampling strategies: optimizing surveillance practices improves diagnostic yield and guides outbreak response more effectively.
- Environmental biosecurity: innovations like virus-inactivating surfaces offer passive defense mechanisms in high-risk environments.
- Feed-related risks: understanding viral persistence in feed leads to improved biosecurity and transport practices.
- Digital technologies: online behavior analysis provides promising support for early outbreak detection, particularly in under-resourced areas.
- Spatial modeling: mapping high-risk areas enables data-driven targeting of interventions, improving the cost-effectiveness and success of control strategies.
- Veterinary mental health: the emotional burden on frontline responders needs greater attention in disease response planning.
- Ecological and genetic complexity: a deeper understanding of wildlife reservoirs and viral genomics is vital for developing long-term control strategies.

The global fight against ASF requires collaboration across disciplines—virology, epidemiology, animal welfare, psychology, data science, and policy. No single approach can solve this challenge. Success will depend on integrating scientific advances with practical tools, stakeholder engagement, and shared international responsibility.

## Final remarks

The articles collected in this Research Topic offer valuable scientific contributions that reflect both the scale and diversity of efforts underway to combat ASF. The research demonstrates that while ASF remains a formidable opponent, progress is being made on multiple fronts. Each study adds a piece to the larger puzzle of ASF prevention, detection, control, and, eventually, eradication.

